# HIV Epidemic in Libya: Identifying Gaps

**DOI:** 10.1177/23259582211053964

**Published:** 2021-11-29

**Authors:** A. Hamidi, P.R. Regmi, E. van Teijlingen

**Keywords:** HIV, libya, women, stigma

## Abstract

**Background:**

HIV (human immunodeficiency virus) and AIDS (acquired immunodeficiency syndrome) became a public issue in Libya after the infection of 400 children in El-Fatih Hospital in 1988. Due to the civil war, social and religious barriers, HIV prevalence is hard to establish, but it is generally believed to be increasing.

**Objective:**

This review (a) assesses the size and scope of the available literature on the HIV epidemic in Libya; and, (b) identifies the nature and extent of research conducted to date.

**Methods:**

A comprehensive search was performed using PubMed, Medline, Web of Science, ScienceDirect, Scopus, Academic Search Ultimate, Cochrane Library and Google Scholar. Primary research studies and official reports that are exclusively on Libya published during 1988−2021 were considered.

**Results:**

In total 25 studies were included: Ten primary research studies, four online news articles, six Government reports, one letter to the editor, one manuscript, three online databases

**Conclusion:**

Despite the low-quality data, the literature suggests there is an increase in HIV infection rates in Libya. Culturally sensitive research on sexual activities, women, HIV preventative methods and attitudes of the Libyan public will assist in developing an effective National AIDS Programme, reducing HIV stigma, supporting People Living with HIV (PLHIV) and decreasing infection rates.

## Background

According to the United Nations, the Middle East and North Africa (MENA) region has the lowest prevalence of HIV (human immunodeficiency virus) and AIDS (acquired immunodeficiency syndrome) in the world, less than 0.1%^1^ yet, the number of new infections is increasing steadily^2^ and it is estimated that there were 20 000 new HIV infections in 2019, a 25% increase from 2010.^3^ The region also has high AIDS-related mortality rates, with 9800 people dying of AIDS-related illnesses in 2018.^4^

It is believed that the reason for the low HIV prevalence is due to the MENA region being predominately made up by countries that are religiously and culturally conservative.^2^ One of these countries is Libya, which is considered as one of the more conservative Arab countries.^5^

A letter to the editor of *The Journal of Infection* in 1988 reported on a study which screened 2064 Libyans (female and male) in 1986 to 1987, in which all samples were negative for HIV antibodies.^6^ The same author continued this study in 1991, testing 10 000 specimens and still found no HIV cases.^7^ This was the first time that HIV was brought to Libyan researchers’ attention.

Daw et al.^8^ conducted a study that assessed HIV infection clustering and trends in Libya over a twenty-five-year period: 1993 to 2017, there were already 975 known cases of HIV in Libya between 1993 and 1997.^8^ However, it was only in 1998 that HIV and AIDS became a public issue in Libya when the government arrested five Bulgarian nurses and a Palestinian doctor accused of deliberately infecting over 400 children with HIV-infected blood, causing an epidemic at El-Fatih Children's Hospital in Benghazi. In response to the outbreak and with the support of the European Union (EU), the Libyan Government established a National AIDS Programme (NAP) in 2002.

Over the span of ten years, despite several attempts to develop a national strategy with the aid of eight million Euro grant^9^ as well as extensive research on the epidemiology of HIV infection in the country and consultancy services from the Liverpool School of Tropical Medicine (LSTM), a strategy is yet to be developed.^10^

The primary reason for the delay is due to the 2011 Libyan Civil War, which led to the HIV response budget as well as the projects planned in cooperation with international partners being postponed.^11^ There was a glimpse of peace and security when the transitional government was installed in December of that year. Under the then Minister of Health, Dr Fatima Elhamroush, HIV became one of its three highest priorities and a comprehensive programme to respond to the growing HIV epidemic resumed.^12^

The National Transitional Council was dissolved in 2012 and to this day, Libya remains in political turmoil which has affected its HIV response planning. This stagnation was acknowledged in the 2015 Country Progress Report, commenting that very little progress was achieved regarding the status of the epidemic and gathering up-to-date data.

Unfortunately, even though there was a genuine attempt to respond to the HIV epidemic, the on-going unrest in the country has impeded the development of a comprehensive NAP and detrimentally, any further research. Our review of the literature on HIV in Libya is timely with the new unified Government in 2021.

The aim of this study is to review published evidence on HIV prevalence, risk factors and social stigma associated with the virus in Libya. Past literature has discussed specific aspects of HIV; however, no study has compiled the results of studies that focused solely on Libya, giving an overarching perspective of the epidemic in the country. Such information should help identify gaps in the literature and provide insight for further research which is essential in developing an effective and comprehensive NAP. Without developing new epidemiological studies, it would be very difficult to develop effective future care plans and measure the HIV and AIDS burden on the country.

## Method

A comprehensive search was performed using PubMed, Medline, Web of Science, ScienceDirect, Scopus, Academic Search Ultimate, and Cochrane Library. An initial search using key terms and medical subject headings (MeSH) was conducted. Search terms included “Libya”, “HIV”, “AIDS”, “HIV-1” and related terms.

Further searches using Google scholar and the reference lists of key articles identified in the primary search were also conducted to determine additional literature.^13^ Primary research studies and official reports that are exclusively on Libya published during 1988−2021 were considered.

Seventeen articles dating from 1988 to 2020 were read and assessed for this review, out of which 10 primary research studies were included and seven were excluded as they focused on physical manifestations of the HIV virus, best practices by health care workers and the benefits of health education intervention for premedical students. As the El-Fatih HIV outbreak of 1988 was an isolated case, material that is related to it as well as the legal proceeding against the nurses and doctor have been excluded as they do not provide a broad representation of the overall epidemic. Online newspapers and magazine articles were included to enhance and provide further details of events and experiences. English and Arabic language material were reviewed.

In total 25 studies were included: 18 publications provided HIV prevalence, 17 showed infections associated with injecting drugs and sexual transmission, one listed marriage as a potential risk factor, five provided limited data regarding women, three research papers explored stigma and attitudes. Three included research on condom use, one researched HIV prevalence in Men who have Sex with Men (MSM) and Female Sex Workers (FSM).

Ethical approval or informed consent was not required for this scoping review which does not involve human participants.

## Results

### HIV Prevalence

Studies and reports dating from 2001 to 2020 identified the HIV prevalence and the number of registered PLHIV (People Living with HIV) in Libya as relatively low. It is assumed that these figures are for Libyan nationals, unless explicitly stated otherwise.

Much of the research on HIV in Libya was conducted between 2004 to 2020 and most studies published similar HIV prevalence rates and number of cases. They show an increase of HIV prevalence from 0.13% in 2004^14^ to 0.2% in 2019.^15^

The number of reported HIV cases: 10 557 in 2000,^10^ 7000 in 2002.^16^ In 2005, 8654 cases were registered,^11^ this increased again to 10 000 in 2006^17^ and 10 450 in 2007.^18^ Daw et al.^8^ reported a total of 8486 registered PLHIV between 1993 to 2017.

As for Women Living with HIV (WLWH), the available data show a steady increase in new infections. It was found that during 1993 to 1997, out of the 975 cases, 22.3% (n = 217) were female and in 2013 to 2017 out of 2147 again 22.3% (n = 497) were female.^8^

It is apparent that there are challenges with gathering national statistical data. Availability and accessibility of any reports on HIV and AIDS in Libya is limited and the handful of reports that are available include outdated and missing data.^10,11^ Referring to newspaper articles for further clarification, it was found that there are inconsistencies with the number of cases disclosed.

The director of the AIDS and STD (Sexually Transmitted Disease) Department stated in 2013, that the number of HIV cases was 12 000 (1990 −2013). This is the same statistics that were announced in June 2012 and officially published in September 2012.^19^

It was reported that HIV cases increased from 6000 in 2017 to 10 000 in 2018,^20^ of whom 25 to 30% were women. In November 2020, the number of people with HIV (excluding the eastern part) was around 6,000, of whom 35 to 40% were women.^21^

Epidemiological data on AIDS related deaths are also scarce, however the recent Libya Country Report-2020^22^ disclosed that the number of people who died of AIDS-related illnesses in 2019 has nearly doubled since 2016 (n = 121). This increase is a consequence of the on-going conflict which has led to the collapse of a historically incapacitated health system, limiting the supply of antiretroviral medicines.^23^

### Risk Factors

The mode of HIV transmission in Libya has shifted throughout the years. It was initially found that most PLHIV were infected via blood or blood products during 1993 to 1997. By 2007, there were no further cases linked with blood.^8^

In 2003, as cited in *HIV*/*AIDS in Libya*,^18^ the Ministry of Health stated that more than 90% of HIV infections are the result of contaminated needles. It identified Sub-Saharan African lorry drivers and immigrants as the original carriers and transmitters of the virus, although there is no conclusive data on the epidemic among African immigrants in Libya and its impact on Libyan nationals.^24^

The HIV prevalence among injecting drug users (IDUs) in Libya is significantly increasing over time; it was 20% (n = 195) during 1993 to 1997 and reached over 50% during 2008 to 2017 (n = 963).^8^ In 2012, HIV prevalence among IDUs was at a startling 87%, (out of a sample of 328), the highest rate recorded anywhere in the world.^25^

Despite the lack of data and reliable evidence on Libya's HIV epidemic, the majority of experts still speculate that injecting drugs is the predominant mode of transmission however, Government sources are indicating that there is an increasing trend towards sexual transmission.^10^ This trend is supported by the Spatiotemporal Analysis and Epidemiological study^8^ that demonstrated infected cases from sexual activities had increased steadily, reaching 40% (n = 858) between 2013 to 2017.

The only study that looked into HIV prevalance in MSM and FSW found that the HIV prevalence in MSM is 3.1% and 15.7% in FSW.^26^ The study also touched upon the potential cross-infection of the HIV virus into the general population. Out of the sample size of 227 MSM and 69 FSW in Tripoli, only 21% of MSM used a condom during last anal intercourse. 63.4% of FSW reported regular condom use during sex with one-time clients and 56.8% with regular clients. However, only 12.1% of MSM and 49.2% of FSW knew how to use condoms correctly. 68.5% of MSM also had sexual intercourse with a female partner in the past and 53% had intercourse with a woman in the last six months.

Mirzoyan et al.^25^ found that only two-thirds of the 75 IDUs in their study, who reported having had sex during the previous month, used a condom. Another study with final year university students (BSc level) reported that only 39% (156 of 400) participant believed that condoms are important for the prevention of HIV transmission.^27^

When researching HIV-related hospitalisations, out of 227 people hospitalised with HIV, most of the female HIV patients were married 57.5% (n = 23) or widowed 22.5% (n = 9) and 87.5% (n = 35) identified martial sexual relations as a route of HIV transmission.^28^

In 2012, a study researching the prevalence of HIV in prisoners in Libya raised the concern for potential for cross-infections, whether through sharing needles or sexual activity.^29^

### Stigma and Attitude

Merely three studies^17,27,30^ were explicit in researching stigma and attitudes towards PLHIV and they focused on Libyan students and dentists.

A total of 1082 high school students from five cities in the North-West of Libya were recruited for a self-administrated questionnaire study (2004 to 2005). The study indicated a high level of stigma towards HIV-infected individuals with 34% (n = 371) strongly agreed and 27% (n = 295) of the students agreed that an HIV infected individual is dangerous to others.^17^

This negative attitude towards PLHIV is shared with final year medical sciences university students. 66% of the 400 final year (BSc) university students agreed that an HIV or AIDS patient should be isolated.^27^ However, a study in 2015 to assess ‘Knowledge and attitudes of Libyan dental students about HIV/AIDS infection and HIV-positive patients’,^30^ concluded that Libyan dental students have a positive attitude towards HIV-positive patients. Around 90% of the 101 students thought that HIV positive patients should be helped, supported and treated but the study also found that 77.7% said that they would inform the patient's close relative about the status of HIV positive patient.

Although the 2015 study could be taken as an optimistic indication of decreased stigma towards PLHIV, it also raises the concern of patient confidentiality and reinforces the fear of ‘people finding out’. A retrospective analysis of HIV-related hospitalisation at Tripoli Medical Centre in 2013 found that a significant number of patients denied any HIV risk factors and others displayed advanced stages upon admission.^28^ It is not determined whether this denial is due to the lack of knowledge or stigma or perhaps both.

## Discussion

Having reviewed all accessible, published materials on the HIV epidemic in Libya, its prevalence and risk factors, the key finding was the overall lack of research on the topic, especially regarding women and sexual transmission. There is also a void when it comes to the stigma PLHIV face in the country, the views, attitudes and knowledge among the general population.

Libya does not have adequate monitoring systems and relies on data received through mandatory screening at various institutions for when certificates are required as well as cases reported by hospitals. This current system offers variable degrees of accuracy and potentially a misrepresentation of the number of PLHIV, who do not know their status as they never needed to be tested, those who choose not to disclose it, or those who are aware of their status and forge certificates of clean health in fear of the repercussions.^19^

There is a recurring theme of inaccurate and out-of-date data within the studies and reports. In HIV/AIDS in Libya,^18^ the author refers to the contrast between the infection rates published by the Libyan Government and those of other independent sources.

Historically the policy of the former regime was not to disclose actual data regarding the virus as part of ‘national security’. Not only was HIV not deemed a priority during Ghaddafi's regime but he went as far as refuting all medical research and evidence, declaring in 2003, that homosexuality was the only cause of HIV and rejecting the notion that HIV can be transmitted through unsafe sex and drug abuse.^19^

Today, in addition to the civil war, the prevalent association between HIV and immoral practices and the fear of the social labelling of being infected with the virus, is the biggest obstacle in Libya. Many might choose to risk their health rather than getting tested and knowing their status for the sake of their own and their family's reputation and honour.

The social stigma is worse than the illness itself, as confessed by PLHIV. Stories of being outcasted by family and community, isolated from society, unable to access healthcare, study or work, are some of the difficulties that PLHIV face in Libya. Society's ignorance and its stereotypical view of the virus is even more damaging and shameful for Women Living with HIV (WLHIV). Girls and women who are found to have contracted HIV are subjected to violence, humiliation, isolation, and invasive virginity tests.^19^

In an attempt to reduce stigma and encourage testing, a National Committee for Reduction of Stigma and Discrimination was formed, mobile VCT (Voluntary Counseling and Testing) services and an HIV & Hepatitis Hotline were introduced. Prevention efforts prior to the conflict were focused primarily on raising awareness of HIV among the general public through the media, training programs for selected professions (eg members of the judiciary, medical providers, teachers and religious leaders) and World AIDS Day celebrations.^10,22^

The body of literature available honed their focus on IDUs as the high-risk group, rather than investigating other HIV risk activities, which might have led to under-representation of some HIV risk activities previously reported in Libya. The literature dictates that the main cause of HIV infections is injected drug and building on this, the Libyan National Strategy has focused on IDUs by collaborating with international experts in training medical staff and initiating further research studies.^22^ However, could it be possible that there might be a religiously or culturally enforced downplay of the significance of other HIV- risk activities.

Islam is a powerful force in society and family dynamics in Libya. It has been suggested that Islam does play an important role in promoting low–risk behaviour^31^ and its greatest contribution to the epidemic is prevention. On the other hand, Islamic values depict sex outside of marriage, homosexuality and use of any intoxicants (alcohol and illegal drugs) as sinful and therefore carry a high-level stigma. Consequently, this prevents those at risk from seeking information, testing, and treatment.^32^

Religious interpretations and culture play a large part in the Government and its respective departments’ approach to HIV. They are under pressure to adhere to what is deemed religiously and culturally appropriate in fear of being accused of contributing to the spread of the disease and condoning immoral behaviour.^19^

There is clear evidence that cross-infection of HIV is occurring through sexual activities and although this is mentioned in various studies and reports, a handful conducted any research in this area. Only two studies^25,26^ researched high-risk groups’ sexual activities. ElFituri^27^ touched on condom awareness and Shalaka et al.^28^ identified marital heterosexual intercourse as a route of HIV infection in women. The most obvious reason for this shortcoming is the weight of religious and cultural sensitivities and potential repercussions.

### Strengths and Limitations

This is the first review to organise and summarise HIV information from published studies that are exclusively on the HIV epidemic in Libya, without any date restrictions. Nevertheless, there were some limitations; firstly, only published literature that's available online was included and it is possible that there are other studies which are not accessible online. Although there were no language restrictions placed, no study on HIV in Arabic was found. Reports, newspaper and magazine articles, which could potentially show bias, were included as it was felt that they provided further evidence and understanding on the HIV epidemic, its impact on the Libyan population and the challenges faced with surveillance and management.

## Conclusion

This review of the literature on HIV prevalence, risk factors, stigma and attitude in Libya found that there is an absence of research into the virus in the country. This is particularly true regarding the increasing HIV infections among women and in particular, married women, in recent years. Religion and culture play a significant role in Libya and therefore may impact research efforts. With that in mind, more diverse research is required to gain a better understanding of the epidemic to be able to control the infection rate and provide PLHIV a dignified life.
